# Analysis of Wine-Producing *Vitis vinifera* L. Biotypes, Autochthonous to Crete (Greece), Employing Ampelographic and Microsatellite Markers

**DOI:** 10.3390/life13010220

**Published:** 2023-01-12

**Authors:** Evangelia V. Avramidou, Ioannis Masaoutis, Theodora D. Pitsoli, Aliki Kapazoglou, Maria Pikraki, Emmanouil A. Trantas, Michael Nikolantonakis, Andreas G. Doulis

**Affiliations:** 1 Hellenic Agricultural Organization ELGO “DIMITRA” (ex. NAGREF), Institute of Mediterranean Forest Ecosystems, Terma Alkmanos, Ilissia, 11528 Athens, Greece; 2 Institute of Olive Tree, Subtropical Plants and Viticulture (IOSV), Laboratory of Plant Biotechnology & Genomic Resources, Hellenic Agricultural Organization ELGO “DIMITRA” (ex. NAGREF), Kastorias 32A, 71307 Heraklion, Greece; 3 Winery of Agrunion of Heraklion, Inatou 32, 71303 Heraklion, Greece; 4 Institute of Olive Tree, Subtropical Plants and Viticulture (IOSV), Department of Grapevine, Hellenic Agricultural Organization ELGO “DIMITRA” (ex. NAGREF), Lykovrissi, 14123 Athens, Greece; 5 Department of Agriculture, Laboratory of Biological and Biotechnological Applications, Hellenic Mediterranean University, 73133 Heraklion, Greece

**Keywords:** *Vitis vinifera* L., SSR markers, Crete, ampelographic descriptors

## Abstract

*Vitis vinifera* ssp. *vinifera* (domesticated grapevine) includes thousands of cultivars, which are classified according to their main uses, as wines, fresh fruits or dried raisins and sultanas since ancient times. Evidence showed that Crete grapevine cultivars and winemaking date back to 2300 BC. In this study, fifty-one genotypes belonging to seven different traditional *Vitis vinifera* cultivars, presumed autochthonous to the island of Crete, were selected for their wine-producing potential and classified by 51 ampelographic descriptors. In addition, five genotypes belonging to two non-autochthonous cultivars were included as out-group controls. Subsequently, in order to characterize genetic diversity, establish genetic relationships within and between cultivars and solve accession-labeling problems, genotypes were fingerprinted employing Simple Sequence Repeat (SSR or microsatellite) markers. Four of the autochthonous cultivars namely ‘Vidiano’, ‘Vilana’, ‘Plyto’, and ‘Moschato Spinas’ are used in the local economy for blanc (white) wine production while the rest, namely ‘Kotsifali’, ‘Liatiko’ and ‘Mantilari’ for Noir (red) wines. The two cultivars employed as out-group were ‘Moschato Samou’ and ‘Moschato Alexandrias’: both white wine producers. Ampelography-based clustering grouped the majority of genotypes along cultivar-specific clusters. All three Moschato cultivars formed a distinct clade pointing to the non-autochthonous origin of ‘Moschato Spinas’. A total of one hundred and thirteen (113) SSR alleles were amplified from thirteen (13) SSR loci, with an average number of alleles per locus equal to 10.23 revealing ample genetic polymorphism. The cumulative probability of identity was also quite high (3.389 × 10^−16^). The overall observed heterozygosity was 0.837 while for twenty-nine of the examined genotypes, at least one private SSR allele was detected. The majority of genotypes were grouped in cultivar-specific clusters. The results of this paper pave the way for the certification and registration of clones of some of the most important wine-producing cultivars in Crete.

## 1. Introduction

In the framework of regional agricultural economies, the selection of locally adapted cultivated varieties (CVs), with appropriate qualitative and organoleptic characteristics, contributes to the quality and unique identity of the final local products. Since the grapevine is a perennial plant with a productive life spanning across a few decades it is of importance to commit resources early by properly selecting propagation materials complying with modern agriculture and current consumer demands [1,2,3].

*Vitis vinifera* cultivars were primarily discriminated by ampelographic approaches. In Greece, traditionally ampelographic descriptors, which are based on the comparison of their morphology were used [4,5] until the discovery of DNA-based markers such as Random Amplified Length Polymorphism (RAPD [6]), Inter Simple Sequence Repeats (ISSR, [7]) and Simple Sequence Repeats (SSR, [8,9,10]). SSR markers have become the preferred markers for the standardization and analysis of genetic variation with regard to grapevine genetic resources, as numerous studies prove [2,3,9,10,11,12,13,14,15,16,17,18,19,20,21,22,23,24,25,26,27,28,29,30,31,32,33,34]. Studies use SSR in order to distinguish grape cultivars, for example, in Cyprus molecular genotyping analysis using 11 SSR markers allowed the accurate identification and discrimination of a set of autochthonous cultivars, clarifying their relationship with Greek, Bulgarian, and western European Vitis genetic material [13]. Similarly, DNA typing at 13 SSR loci identified 28 different genotypes comprising mainly indigenous germplasm cultivated in the archipelago of Malta [14] contributing to the accurate identification of unknown or neglected grapevine germplasm in the region. Genotyping with 23 SSR markers assessed the genetic diversity of Moroccan grape accessions as compared to the Maghreb and European gene pools [15]. Sardinian grapevine cultivars were genotyped with SSR markers towards accurate identification of indigenous cultivars, resolving issues of synonyms/homonyms and false name assigning [16]. Other studies completing this scope can be also mentioned such as [17] where 411 accessions of the Grapevine Germplasm Bank were assessed using 26 SSR markers in Spain. SSRs remain the markers of choice in recent years, e.g., in 2022, in Argentina discrimination of cultivars was accomplished with SSR markers [18].

In Greece, about 400 grapevine cultivars have been recorded, many of which are considered autochthonous while at least 36 of them are believed to be of Cretan origin [20,35,36]. The definition “autochthonous” originates from two ancient Greek words: αυτός = he, she, it and xθών = land. Autochthonous cultivars are cultivars that are indigenous—native to the origin where they were found. The need to properly identify and discriminate the plethora of Greek grapevine cultivars and address issues of varietal misnaming (synonyms/homonyms) has led to a series of studies, in recent years that employ a combination of phenotypic and genetic characterization of grapevines from cultivation centers and ampelographic collections. Particularly on the island of Crete (Greece), where viticulture has been practiced over the last few millennia, there is an ongoing effort for the introduction of standardized autochthonous plant material in local commercial viticulture [20,36,37]. This material has been maintained and bred traditionally, on a farm, but only recently it became the focus of specific ampelographic and molecular analysis [38]. Such local materials are expected to be more suitable for organic production since they were not selected for the conventional high-input sector and are expected to contribute to the product differentiation and branding of locally produced wines and table grapes. A very recent study reported comprehensive molecular fingerprinting of a large collection of wine and table grapevine cultivars from different vine-growing areas of the island of Crete [20]. Employing 13 SSR markers this study revealed the genetic relationships and population structure among 44 local cultivars collected from different sites on the island and presented a first proposal with regard to their ancestry.

Moreover, in order to adequately discriminate cultivars of Greece, a combined analysis of ampelographic descriptors (ampelographic descriptors are based on the comparison of their morphology [21]), oenological traits, and genotyping with SSR microsatellite markers were used to study a series of native grapevine varieties from Greece [11]. Combinatorial analysis utilizing ampelographic and RAPD molecular markers led to the identification, discrimination and genetic analysis of 49 grapevine cultivars from Northern, Western and Central Greece collected from productive vineyards in cultivation centers [39]. Ampelographic and genetic characterization of biotypes and variants of the grapevine cultivar ‘Korinthiaki Staphis’ and of the ‘Mavroudia’ group of grapevines cultivated in Greece also have been reported recently [36]. Furthermore, ampelographic and SSR markers were employed in order to assess the genetic relatedness among 12 indigenous grapevine varieties originating, mainly, from northern and western Greece [20]. Additionally, a recently published study for northern Greece combined ampelographic traits and microsatellite markers to study the genetic diversity within and among 96 grapevine genotypes belonging to 36 *V. vinifera* subsp. *vinifera* cultivars, predominantly representing autochthonous landraces [2].

In addition, as molecular approaches are continuously evolving, next-generation sequencing also used and identified millions of Single Nucleotide Polymorphisms (SNP) and a number of insertion/deletions (indels) differentially distributed along the genomes of four widely cultivated Sardinian grapevine cultivars that may reflect the phenotypic differences observed amongst them [40]. Likewise, comprehensive molecular characterization employing both SSR and SNP markers evaluated the genetic diversity and population structure of a large grapevine germplasm collection from Italy comprising cultivated grapevines, wild individuals, interspecific hybrids and rootstocks [22]. Genotypic analysis with a large number of SNP markers assessed the genetic diversity of Tunisian wild and cultivated grapevine genotypes and suggested an origin for the Tunisian cultivated germplasm deriving from the introduction of cultivars from other Mediterranean areas rather than from local wild populations [41]. Furthermore, recent genetic analysis of a large number of Sicilian Vitis wild populations and indigenous cultivars with 23 SSR markers revealed a rather close relatedness between wild and cultivated Sicilian varieties pointing to introgression and/or domestication events and the possible contribution of indigenous wild populations to the genetic makeup of local cultivars [23]. Additionally, combining SSR data with genome content could also further contribute to the discrimination of cultivars or landraces as a recent study from [42] pointed out. Additionally, recent studies also pinpoint the need to efficiently combine molecular, morphological and biochemical results in order to discriminate cultivars all over the world [11,27,28,39,43,44] and to protect the Vitis germplasm due to ongoing climatic changes [45].

The aim of the present study was to analyze the genetic structure between as well as within seven multi-genotype cultivars originally selected from autochthonous and traditionally cultivated Cretan grapevines (landraces) employing ampelographic on one hand and molecular markers on the other. SSR genetic fingerprinting of the Cretan grapevine diversity will contribute to the production of certified propagation materials that will, in turn, facilitate the production of quality wines within the framework of certified agriculture. Specific objectives of the present study were to: (i) assess and partition inter- and intra-cultivar genetic variability of selected grape cultivars autochthonous to Crete which are of particular significance to local wine production, (ii) provide a consensus description of traditional genetic materials and (iii) assist with cultivar registration and certification. Towards these aims, the current work involved ampelographic characterization as well as genetic typing employing the co-dominant SSR molecular markers.

## 2. Materials and Methods

### 2.1. Plant Material

Herein, the analyzed grapevine individual genotypes (Table 1) originated from the national germplasm and ampelographic collection (pre-basic grapevine) located at the Messara Agricultural Research Station (locality of Abelouzos, Crete, Greece, geographic longitude 24°56′13″, geographic latitude 35°3′46″) of the National Agricultural Research Foundation (NAGREF; presently Hellenic Agricultural Organization “Demeter”). Following ELISA testing against six major viral pathogens and a formal agreement between NAGREF and local commercial nurseries, these genotypes were released in 2006 for use with the local commercial grape growers. This is the only known case of dissemination of characterized grape genetic materials in Greece. Nevertheless, ampelographic characterization was only completed in 2009. Presently examined biotypes (candidate clones) are still undergoing a full clonal evaluation.

### 2.2. Ampelographic Classification

Observations were taken for two consecutive years at the Ampelouzos collection from individual genotypes while the mean value was employed for subsequent analyses. Initially, eighty-one (81) ampelographic descriptors were evaluated according to the International Organization of Vine (OIV) while only variable ones were retained for subsequent analysis. The Manhattan dissimilarity index was employed to determine relatedness between biotypes pre-assigned to cultivars. Subsequently, the Unweighted Pair Group Method with an Arithmetic average (UPGMA) clustering algorithm was employed for similarity dendrogram construction employing the NTSYSpc (ver. 2.21L) software package [46].

### 2.3. DNA Isolation

Expanding leaves were collected from the Ampelouzos collection, wrapped in aluminum foil, dipped in liquid nitrogen and transferred to −80 °C till further use. Genomic DNA was isolated separately from 56 genotypes, considered representatives of each cultivar, using the Plant Mini Kit (Qiagen, Germantown, MD, USA) according to the manufacturer’s instructions. For the initial grinding, the automated mill Tissuelyser (Rietchke-Qiagen, Maryland, USA) was employed in combination with liquid nitrogen. Once eluted, DNA was stored at 4 °C until further use. DNA was quantified employing the Hoechst 33,258 fluorescence dye (Sigma, St. Louis, MO, USA, No. B2883) on a computerized TD 700 fluorometer (Turner Designs, Sunnyvale, CA, USA) against calf thymus DNA standards (Sigma, No. D4764).

### 2.4. Determination of SSR Markers

Thirteen microsatellite loci were investigated: VVMD5, VVMD7 [10], VVMD27 [47], VVS2 [48], VRZAG62, VRZAG79, VRZAG64, VRZAG83 [8], VRZAG21, VRZAG47 [49], VVUCH11, VVUCH12, and VVUCH29 [25]. The first six loci are adopted by OIV and by the European Vitis Database (http://www.eu-vitis.de/index.php (accessed on 11 November 2011) as part of a common set of SSR descriptors. The rest of the loci were selected on the basis of published polymorphic information content on genetic materials of Cretan geographic origin [38]. Microsatellite polymorphisms were detected by labeled forward primers on an automated sequencer. Polymerase Chain Reactions (PCR) were carried out in 20 μL final volume using a Perkin Elmer 9600 thermocycler. PCR reactions consisted of 25 ng of template DNA, 0.2 mM of each dNTP, 0.2 μM of each primer, 2.5 mM MgCl_2_ and 1 U of RedTaq DNA polymerase (Hytest, Turku, Finland). PCR reactions were performed at an initial denaturation for 5 min at 95 °C, followed by 35 cycles consisting of 95 °C for 30 s, the corresponding annealing temperature for 45 s, and 72 °C for 45 s. At the end, a final extension of 72 °C for 10 min was performed. PCR products were analyzed on an ABI 3730xl (Applied Biosystems, Waltham, MA, USA) automated fluorescence sequencer. For data scoring the Genemapper 4.0 (Applied Biosystems, Waltham, MA, USA) software was used, by employing LIZ 500 as a size standard. The matrix produced was used for all subsequent statistical and cluster analyses.

### 2.5. Statistical Analysis of SSR Markers

GeneAlEx ver. 6.5 was used, as a plug-in module within Microsoft Excel [50], for the determination of the number of private and total alleles by locus, the effective number of alleles (Ne) and the Analysis of Molecular Variance (AMOVA). The Cervus 3.0 software [51] was used for the estimation of expected heterozygosity (He), observed heterozygosity (Ho), probability of identity (PI) and probability of null alleles. The similarity matrix was produced employing the Lynch distance metric [52]; a simple band-sharing measure [termed “band” similarity coefficient within the NTSYSpc (ver. 2.21q) software package [46] while the similarity dendrogram was constructed by the UPGMA algorithm employing NTSYSpc.

The matrices produced from the ampelographic data (based on the Manhattan dissimilarity index) and from the SSR (based on the Lynch “band” similarity index) were tested for their degree of congruence using the two-way Mantel test (Mantel 1967) employing the Matrix Comparison (MXCOMP) procedure in NTSYSpc (normalized statistic) and 1000 permutations.

## 3. Results

Fifty-one (51) autochthonous Cretan along with five (5) out-group genotypes, were typed employing ampelographic description, and SSR markers. Cluster analyses involved dendrogram construction by including both white and red berry genotypes. The number of alleles per marker, He (expected heterozygosity), Ho (observed heterozygosity), Probability of Identity (PI) and Probability of null alleles are presented in Table 2.

### 3.1. Ampelographic Classification

**Figure 1:**
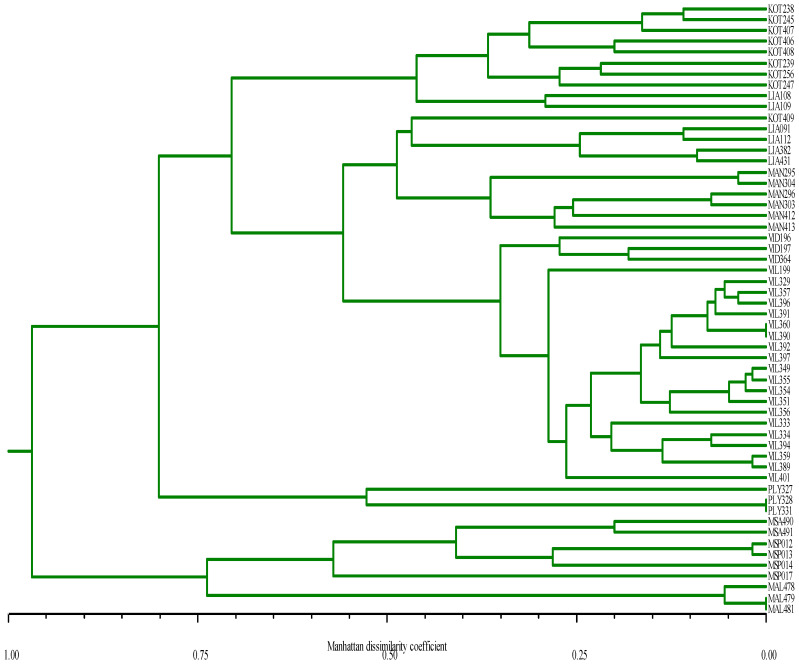
UPGMA dendrogram of 56 *Vitis* genotypes employing ampelographic data (55 descriptors) and the Manhattan dissimilarity coefficient. Herein we can discriminate different groups for example two groups of ‘Liatiko’ (red berry) were formed. One group consisted of two genotypes linked with the ‘Kotsifali’ group while the other consisted of four genotypes linked with the ‘Mandilari’ group.

The data from morphological (ampelographic) characterization were used to construct a dendrogram for the partitioning and representation of the inter- and intra-cultivar variability (Figure 1). In total, 81 ampelographic descriptors were collected (data not shown). Nevertheless, only informative (variable) ones were retained for subsequent cluster analysis. Specifically, 55 descriptors were found informative with the white-berry cultivars (‘Vidiano’, ‘Vilana’, ‘Plyto’, ‘Moschato Spinas’, ‘Moschato Samou’) while 45 of these descriptors were also found variable within the red-berry cultivars (‘Kotsifali’, ‘Liatiko’, ‘Mandilari’). Eventually, cluster analysis involved the entire set of genotypes tested (56) while it employed the 55 ampelographic descriptors originally identified as informative within the white-berry cultivars (Figure 1). Prima facie, each of the investigated genotypes was considered as a genetically independent individual. Ampelography-based clustering grouped most of the genotypes (53 out of 56) along cultivar-specific (monophyletic) majority clusters. Cultivars appeared as monophyletic groups were: ‘Mantilari’ (MAN), ‘Vidiano’ (VID), ‘Vilana’ [24], ‘Plyto’ (PLY), ‘Moschato Alexandrias’ (MAL) and ‘Moschato Samou’ (ΜSA). Two noir cultivars, ‘Liatiko’ (four out of six genotypes) and ‘Mandilari’ (all six genotypes) and one Kotsifali are clustered together with twenty (20) ‘Vilana’ (a blanc cultivar) genotypes and three ‘Vidiano’ (a blanc cultivar) genotypes. Interestingly, two ‘Liatiko’ genotypes (LIA108, LIA109) form a common sub-group together with eight out of nine ‘Kotsifali’ genotypes. Inversely, the remaining ‘Kotsifali’ genotype (KOT409) forms a separate sub-group together with the remaining four ‘Liatiko’ genotypes. From the three red (noir) cultivars, only ‘Mandilari’ (with all six individual genotypes) appears as a truly monophyletic group. With blanc cultivars all three genotypes of ‘Plyto’ form a monophyletic group while two of them (PLY328 and PLY331) appear ampelographically identical. ‘Plyto’ is further differentiated from all the above cultivars. On the other hand, all three Moschato cultivars (blanc) form a distinct cluster clearly differentiated from all the above clusters. Within this Moschato cluster, ‘Moschato Alexandrias’ appears as a cultivar-specific sub-group while two (MAL479 and MAL481) out of three genotypes are ampelographically indistinguishable. Four genotypes of ‘Moschato Spinas’ show high ampelographic similarity among themselves while they form a common group with the two genotypes of ‘Moschato Samou’. In addition, three cases of ampelographically identical clones, existing within the same cultivar, were observed (i) VIL360, VIL390, (ii) PLY328, PLY331 and (iii) MAL479, MAL481.

### 3.2. SSR Analysis

Within the presently analyzed genetic pool, all thirteen loci were found to be highly polymorphic. Table 2 indicates the sizes of amplified alleles for each locus and each cultivar separately. Private alleles are also highlighted. Across all analyzed genotypes, the total number of amplified alleles was 133 with an average value of 10.23 alleles per locus, with a range varying from six alleles for locus VRZAG64 to 16 alleles for locus VVUCH29 (Table 2). Averaged values of expected heterozygosity (He) and observed (Ho), for all tested genotypes, were 0.799 and 0.837, respectively. Locus VRZAG62 exhibited the lowest Ho (0.537) while loci VRZAG64 and VRZAAG83 exhibited the highest (1.000). The values of observed heterozygosity were higher than those of expected heterozygosity with seven out of thirteen employed loci. With loci VVMD7, VVMD27, VRZAG62, VVZAG21, VVZAG47, and VVUCH12 the inverse was true and this allowed for increased probability of null alleles (VVMD7, VRZAG62, VVZAG21, values above 0.05; Table 2). The probability of identity (PI) ranged from 0.032 to 0.145; the highest value was provided by loci VVZAG47, VRZAG62 and VRZAG64. However, the probability to find different plants with the same profile when all loci are combined was indeed very low (cumulative PI = 3.33 × 10^−16^; Table 2).

**Figure 2:**
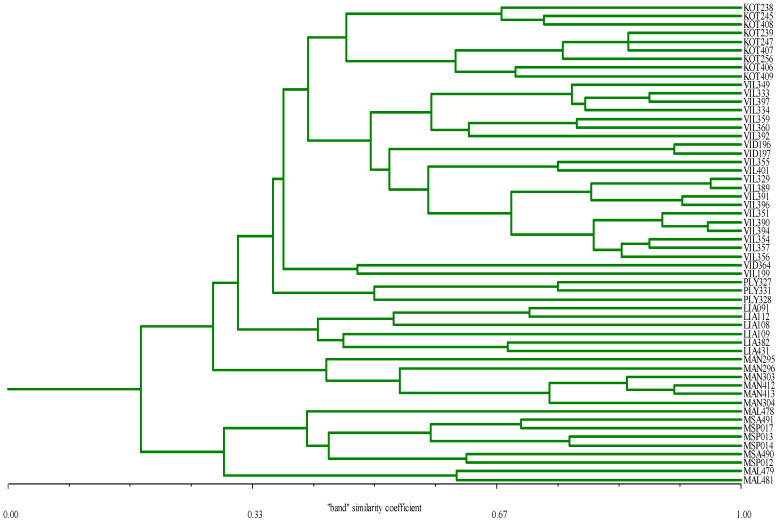
UPGMA dendrogram of 56 *Vitis* genotypes derived from SSR data (13 loci) employing the “band” similarity coefficient. Groups are formed as for example all ‘Liatiko’ genotypes were uniformly grouped into a single group, distantly linked to another group which also included ‘Plyto’, ‘Vilana’, ‘Vidiano’ and ‘Kotsifali’.

Analysis of molecular variance (AMOVA) significantly (PhiPT = 0.359; *p* = 0.001) partitioned genetic variance into two hierarchical levels (Table 3); one among genotypes belonging to different cultivars (among cultivars; 36%, and another among genotypes belonging to the same cultivar (within cultivars; 64%). A UPGMA similarity dendrogram based on SSR data and the Jaccard similarity coefficient is shown in Figure 2. Ample molecular-genetic diversity, both between as well as within cultivars, was revealed by employing thirteen SSR loci. Cultivars ‘Kotsifali’, ‘Plyto’, ‘Liatiko’ and ‘Mantilari’ appeared as monophyletic (cultivar-specific) groups. Nineteen out of twenty genotypes of ‘Vilana’ formed a group that also included two out of three genotypes of ‘Vidiano’. A single ‘Vidiano’ genotype (VID364) formed a separate sub-group together with a single ‘Vilana’ genotype (VID364) further differentiated from the ‘Vilana’-‘Vidiano’-‘Kotisfali’ cluster. All genotypes of the three ‘Moschato’ cultivars cluster along the same broader group. The Mantel correlation coefficient (r) between the ampelography-based structure and the SSR-based structure of analyzed materials was −0.323.

## 4. Discussion

Characterization of multi-genotype autochthonous *Vitis vinifera* cultivars may be a complex task. Within the island of Crete, there is, at present, a wide range of grapevine cultivars being cultivated which are: (i) old/autochthonous, (ii) widespread non-autochthonous and (iii) locally, on-farm selected hybrids, derived from crosses within and between autochthonous cultivars or outcrosses with other non-autochthonous cultivars. In addition, one habit in the local viticultural practice, included for some time, propagation via vegetative materials without any further estimation of genetic variability incurred during this process and resulting in the accumulation of multiple mutations within individual genotypes, which affect, in turn, directly or indirectly, phenotype and the corresponding variability within the cultivars [53]. Furthermore, it is not accurately known how many of the cultivars of a region are unique to this region, and not synonymous with varieties also grown elsewhere in the broader Aegean area. The problem can become more complicated since it can be anticipated that farmers refer to many different cultivars with the same name (homonyms) or they use different cultivar names for the same cultivar (synonyms). In a previous study, [38] employed a single genotype per cultivar, and eleven microsatellite loci and provided a first genetic comparison and molecular classification of Greek (including some Cretan) grape cultivars. The microsatellite profiling of 50 cultivars (out of purportedly 400 Greek cultivars) and a small percentage of genetic resources of grapevine across the entire country of Greece produced 47 single profiles from 50 cultivars and provided discrete information necessary for their differentiation. In a subsequent study, the same authors fingerprinted, with four microsatellite loci, grape individuals growing in vineyards across Crete that were in the stage of initial assignment to different cultivars [25]. The work presented herein only employed biotypes retained for a subsequent stage of clonal evaluation and were all different from the individuals characterized in the preceding study [25]. Further, presently employed biotypes were all characterized with both morphological (ampelographic; 55 in total) as well as molecular (SSR; 13 in total) markers. Present cultivar assignment based on ampelographic data agreed with grouping based on molecular markers on the basis of forming monophyletic or near monophyletic (cultivar-specific) clusters. Nevertheless, topologies between the two similarity dendrograms exhibited some local differences. With the present study, the number of SSR markers employed (a total of thirteen; 13), as well as the degree of SSR polymorphism revealed due to the inherent within cultivar genetic variability allowed for individual genotype discrimination, i.e., clonal differentiation within each cultivar. Despite this, the vast majority of clones (independent genotypes) were clustered along cultivar-specific clusters further adding to the overall validity of (i) initial genotype assignment to cultivars and (ii) the different analytical approaches employed.

In general, ampelographic classification based on OIV descriptors and the Manhattan dissimilarity coefficient resulted in a dendrogram that was in very good agreement with the a priori assignment of genotypes to cultivars since individual genotypes were grouped in cultivar-specific clusters (Figure 1). One important observation produced from the ampelography-based classification was that two groups of ‘Liatiko’ (red berry) were formed. One group consisted of two genotypes linked with the ‘Kotsifali’ group while the other consisted of four genotypes linked with the ‘Mandilari’ group. This type of re-grouping (i.e., of ‘Liatiko’ with either ‘Kotsifali’ or ‘Mandilari’) was not confirmed by the grouping based on genetic distances established via SSR markers (Figure 2). According to the SSR dendrogram, all ‘Liatiko’ genotypes were uniformly grouped into a single group, distantly linked to another group which also included ‘Plyto’, ‘Vilana’, ‘Vidiano’ and ‘Kotsifali’ (Figure 2). In the ampelography-based dendrogram (Figure 1) it could be further observed that all genotypes of Moschato cultivars formed a group that is the most differentiated with respect to all the remaining Cretan cultivars. This clear separation of Moschato genotypes from all the rest is in full agreement with the molecular-genetic picture (Figure 2). In Figure 1 ‘Moschato Samou’ and ‘Moschato Spinas’ appeared closer together with respect to the third muscat cultivar (‘Moschato Alexandrias’) which is in turn distantly associated with the two other muscat cultivars. This relationship among Moschato cultivars is consistent with the classification proposed earlier for Moschato cultivars, utilizing RAPD molecular markers [37].

Regarding the genetic parameters results, all thirteen loci were found to be polymorphic with the total number of amplified alleles and the average value of alleles per locus (10.23) being higher than those found in the literature when a comparable number of SSR loci were used within genetic backgrounds of autochthonous grapes from other regions. Similarly, values of observed as well as expected heterozygosity compared very favorably with published values from autochthonous grapes from other regions. The estimated average value of genetic diversity (expected heterozygosity, He) for tested genotypes was 0.7991 and was lower than this found in northern Greece cultivars by [2] and from three Anatolian locations [54]. Moreover, He was higher than [24], who studied 1378 wild and cultivated grapevines collected around the Mediterranean basin and from Central Asia; higher than [12] who studied 15 Georgian aboriginal cultivars and 42 individuals of wild grapevine from different regions of Georgia and adjacent Turkey, higher than [17] who studied 411 accessions of Spanish Vitis germplasm, higher than [55] where 196 grapevine samples from five countries of the Western Balkan region assessed with SSR markers and also higher than [30] who studied 222 cultivated (*Vitis vinifera*) and 22 wild (*V. vinifera* ssp. sylvestris) grape accessions. Moreover, the estimated value of He was similar to one by [56] for genotypes cultivated in the European regions. The estimated He indicates the cryptic genetic variation that the autochthonous cultivars retained through time. The lower Ho values observed in the sum of the tested genotypes are, most probably, the result of a degree of inbreeding. Furthermore, the cumulative value of the probability of identity (PI) for tested genotypes (3.38 × 10^−16^) was lower than that obtained from [56] but higher than [2,55], indicating that those 13 SSR loci discriminated autochthonous cultivars.

Our findings taken together, corroborate the proposal that hybridization events seem to be constrained by microgeographic and ecological barriers. Nevertheless, human intervention appears to play a role in such a clear maintenance of the barriers observed with the majority of the analyzed cultivars. On the other hand, it appears that molecular differentiation precedes morphological differentiation (i.e., this leads to a new clone formation). This is evidenced by the presence of three couples (‘Vilana’, ‘Plyto’, ‘Moschato Alexandrias’) of ampelographically identical genotypes (see above Results, Ampelographic classification,) which are molecularly quite differentiated (as judged by their respective SSR fingerprints).

### 4.1. In Depth Analysis of Cultivars

Since this is the first time that such a comprehensive presentation of Cretan autochthonous cultivars was conducted we proceed with further discussing each cultivar’s characteristics within the framework of all other presently examined ones.

#### 4.1.1. ‘Vilana’-’Vidiano’

As can be seen in the dendrograms produced by the ampelographic and SSR data, genotypes of ‘Vilana’ were clustered together, whereas ‘Vidiano’ genotypes were associated with ‘Vilana’ either as a separate branch encompassing all three ‘Vidiano’ genotypes (Figure 1; ampelographic) or as a sub-cluster of two genotypes within the ‘Vilana’ cluster (Figure 2; SSR), respectively. As expected, this picture suggests a high degree of similarity between these two cultivars, indicating a common origin. Up to 20 years ago, ‘Vidiano’ was exclusively cultivated in the region of Rethymnon, Crete while ‘Vilana’ was exclusively present in the region of Heraklion, Crete. Given the geographic distance of the two traditional areas of cultivation, it appears that these two cultivars are very similar not due to pollen-mediated gene flow but because they share a common ancestor. Interestingly, in the SSR-based dendrogram, one genotype of ‘Vidiano’ and one of ‘Vilana’ (VID364 and VIL199, respectively) formed a cluster distinct from the major “Kotsifali’—‘Vilana’—‘Vidiano’ cluster (Figure 2).

#### 4.1.2. ‘Plyto’

The traditional cultivation area of ‘Plyto’ was quite extensive, while serious overlap with other cultivars was observed within the cultivation area of this cultivar, especially in the Rethymnon region. In fact, the two cultivars were frequently found together in the same traditional vineyards. It was then possible that the exchange of genetic material took place, thus reducing the genetic distance between them. In the field, there is confusion among grape growers regarding the separation and identification of these two cultivars. From the comparison of ampelographic and SSR data, the linkage of ‘Plyto’ genotypes with ‘Vilana’ and ‘Vidiano’ genotypes to form a separate branch can be linked to a broader group consisting either of ‘Vilana’, ‘Vidiano’, ‘Kotsifali’ and ‘Liatiko’ genotypes in the ampelographic data or of ‘Vilana’, ‘Vidiano’ and ‘Kotsifali’ genotypes in the case of SSR data. Interestingly, the complete ampelographic identity of PLY328 and PLY331 is only coupled with a big high SSR-based differentiation.

#### 4.1.3. ‘Liatiko’

‘Liatiko’ is thought to be an ancient local Cretan cultivar [36]. On the basis of ampelography, ‘Liatiko’ genotypes were not clustered in one group. Instead, two genotypes were clustered together with a predominantly ‘Kotsifali’ cluster while the remaining four ones formed a separate cluster also including one ‘Kotsifali’ genotype. SSR analysis confirmed the high ampelographic variability between ‘Liatiko’ genotypes and permitted their grouping in a monophyletic single cultivar cluster.

#### 4.1.4. ‘Kotsifali’

Similarly, to ‘Liatiko’, ‘Kotsifali’ is also considered an ancient cultivar and is, at present, the most significant wine-producing indigenous red berry cultivar in Cretan viticulture. In the SSR dendrogram, it appeared as a single-cultivar cluster while in the ampelographic dendrogram, there is a major ‘Kotsifali’ cluster with a partial mix-up with ‘Liatiko’ genotypes (see Discussion on “Liatiko” above). Overall, this cultivar exhibits a strong phylogenetic signal with a clear morphological as well as molecular-genetic distinction, further pointing to the specificity of its use, old age, or recurrent selection cycles.

#### 4.1.5. ‘Mandilari’

‘Mandilari’ is also considered an ancient cultivar cultivated in central Greece and the islands of the central Aegean Sea. It is mostly used as a color additive to wine produced from other cultivars, while it is considered to possess high wine-making potential due to its special aroma. ‘Mandilari’ genotypes appeared as single clusters in both marker systems exhibiting a strong phylogenetic signal.

#### 4.1.6. ‘Moschato’

In our study, all Moschato (Muscat) genotypes appeared as a single group following ampelography- as well as SSR-based cluster analysis. According to ampelographic data, the two ‘Moschato Alexandrias’ genotypes appeared as a single sub-group, different from the other sub-group which included all other Moschato genotypes. The latter (‘Moschato Spinas’ and ‘Moschato Samou’) formed two closely linked but separate groups. It can be proposed that ‘Moschato Spinas’ was transferred, during historical times, from the island of Samos to the village of Spina, Chania, Crete. All three different subgroups of the presently examined Moschato family, are linked together and form a larger Moschato cluster indicating that they are indeed closely related genotypes, originating from a common progenitor, probably by the accumulation of mutations [39]. Given that all Moschato genotypes were grouped together and exhibited the least similarity to all other Cretan cultivars it is then proposed that ‘Moschato Spinas’ is not an autochthonous Cretan cultivar. Despite this, it is well-adapted and widely optimized for use with local winemakers.

## 5. Conclusions

Overall, the current study presents a morphological and molecular characterization of different biotypes of traditional Cretan grapevine cultivars and reveals genetic variability not only across cultivars but also among genotypes within the same cultivar. It is apparent from our study, the importance of using both molecular and morphological differentiation in order to distinguish Vitis cultivars. SSR markers provide us the evidence that the presence of three couples (‘Vilana’, ‘Plyto’ ‘Moschato Alexandrias’) of ampelographically identical genotypes are molecularly quite differentiated. To our knowledge, this is the first study to simultaneously characterize an assortment of candidate clones (biotypes) involving the use of a high number of ampelographic descriptors as well as of microsatellite markers (Simple Sequence Repeats; SSRs) for autochthonous cultivars in Crete. The outcome of this study will contribute to the accurate identification of local Cretan grapevine germplasm widely used in winemaking and can serve as a baseline, at the cultivar and clonal (biotype) level, which will facilitate its proper registration and certification.

## Figures and Tables

**Table 1:** Description of 56 wine-producing individual grape genotypes analyzed in the framework of the present study, by berry color, cultivar and genotype code.

Cultivar	Number of Independent Genotypes Analyzed	Genotype Code
Noir (red berry)		
‘Kotsifali’	9	238, 239, 245, 247, 256, 406, 407, 408, 409
‘Liatiko’	6	091, 108, 109, 112, 382, 431
‘Mandilari’	6	295, 296, 303, 304, 412, 413
sub-total Noir	21	
Blanc (white berry)		
‘Vidiano’	3	196, 197, 364
‘Vilana’	20	199, 329, 333, 334, 349, 351, 354, 355, 356, 357, 359, 360, 389, 390, 391, 392, 394, 396, 397, 401
‘Plyto’	3	327, 328, 331
‘Moschato Spinas’	4	012, 013, 014, 017
sub-total Blanc	30	
Total autochthonous	51	
Out-group control	5	
‘Moschato Samou’	2	490, 491
‘Moschato Alexandrias’	3	478, 479, 481
Total analyzed	56	

**Table 2:** Analysis of SSR profiles in 51 Cretan cultivars and five out-group genotypes: number of alleles, (Ne) number of effective alleles, observed (Ho) and expected (He) heterozygosity, probability of identity (PI), and probability of null alleles at 13 nuclear SSR loci.

Locus	Number of Alleles	Ne	He	Ho	PI	Probability of Null Alleles
VVS2	12	3.175	0.794	0.981	0.072	−0.127
VVMD5	9	3.310	0.850	0.893	0.044	−0.030
VVMD7	10	2.793	0.763	0.679	0.082	0.058
VVMD27	12	3.017	0.858	0.839	0.040	0.007
VRZAG62	8	2.385	0.655	0.537	0.145	0.146
VRZAG79	10	3.283	0.844	0.907	0.046	−0.047
VVZAG21	15	3.060	0.869	0.704	0.034	0.099
VVZAG47	9	2.435	0.749	0.732	0.104	−0.000
VRZAG64	6	2.085	0.758	1.000	0.100	−0.154
VRZAG83	9	3.507	0.802	1.000	0.069	−0.125
VVUCH11	7	2.857	0.781	0.870	0.087	−0.059
VVUCH12	10	2.516	0.795	0.764	0.072	0.022
VVUCH29	16	3.502	0.871	0.981	0.032	−0.067
Total	133				3.389 × 10^−16^	
Mean	10.23 (allele/locus)	2.085	0.799	0.837		

**Table 3:** AMOVA summary table for the SSR data and for the seven (7) autochthonous Cretan and two (2) out-group control wine-producing grape cultivars. Probability for PhiPT estimates (*p*-value) is based on 999 permutations across the entire data set.

Source of Variation	Degrees of Freedom	Sum of Squares
among cultivars	8	236,907
within cultivars	47	333,861
total	55	570,768

## Data Availability

Not applicable.
